# Lateral force separation of biopolymers using an atomic force microscope

**DOI:** 10.1063/5.0153116

**Published:** 2023-05-25

**Authors:** Mark S. Anderson

**Affiliations:** Jet Propulsion Laboratory, California Institute of Technology, 4800 Oak Grove Drive, M/S 125-109, Pasadena, California 91109, USA

## Abstract

The lateral force separation of long chain biomolecules is demonstrated using an atomic force microscope (AFM). This is achieved by using an AFM tip to pull molecules away from the edge of a nanofluidic solution. By monitoring the torsion on the AFM cantilever, a characteristic force–distance signal is produced when long chain molecules separate and detach from the solvent edge. This lateral force separation using AFM (LFS-AFM) is demonstrated on egg albumin proteins and synthetic DNA strands. The detected length of the protein and nucleotide biopolymers was consistent with their calculated molecular contour length. LFS AFM provides separation and detection of single polymer strands that has potential applications in biochemical analysis, paleontology, and life detection.

## INTRODUCTION

Atomic force microscopy (AFM) is a fundamental imaging tool for nanotechnology and biology. AFM technology is centered on a sharp tip that is attached to a microfabricated cantilever. The cantilever-tip is raster scanned across a surface, typically using piezoelectric actuators. The sensitive measurement of the cantilever deflection provides topographic information and force measurements between the tip and surface.^1–3^ In addition to providing topographic imaging, the AFM has been modified to acquire localized spectroscopic, chemical, and physical property information.^4,5^

The AFM tip-cantilever mechanism has been further applied to the nanofluidic manipulation of liquids on surfaces. An early application of AFM mediated nanofluidics, is the so-called dip pen nanolithography (DPN) method, that provides removal and deposition of liquids using the AFM tip-cantilever mechanism.^6^ Additional nanofluidic functions have been subsequently demonstrated with the AFM mechanism providing nanofluidic injection, pumping, switching, and detection.^7^ These diverse nanofluidic functions culminated in AFM mediated liquid chromatography that uses the tip-cantilever for shear driven pumping and detection of the separated components.^8^

AFM has also been demonstrated to measure the length of long chain polymers by using the tip to adhere to a molecule and pull molecules vertically off a surface where a force signal is measured upon detachment.^9–12^ This AFM force spectroscopy also detects the intramolecular forces related to the unfolding events of a molecule and the intermolecular forces between two biomolecules.^13^ When molecules are chemically attached at one end to a surface, their length and molecular weight can be determined by the detachment force-distance signal.^14,15^ This has been applied to the analysis of fossils where long chain molecules have been detected. The detection of long chain molecules has been proposed as a generic bio-marker indicator for life detection.^16^

**FIG. 1.**
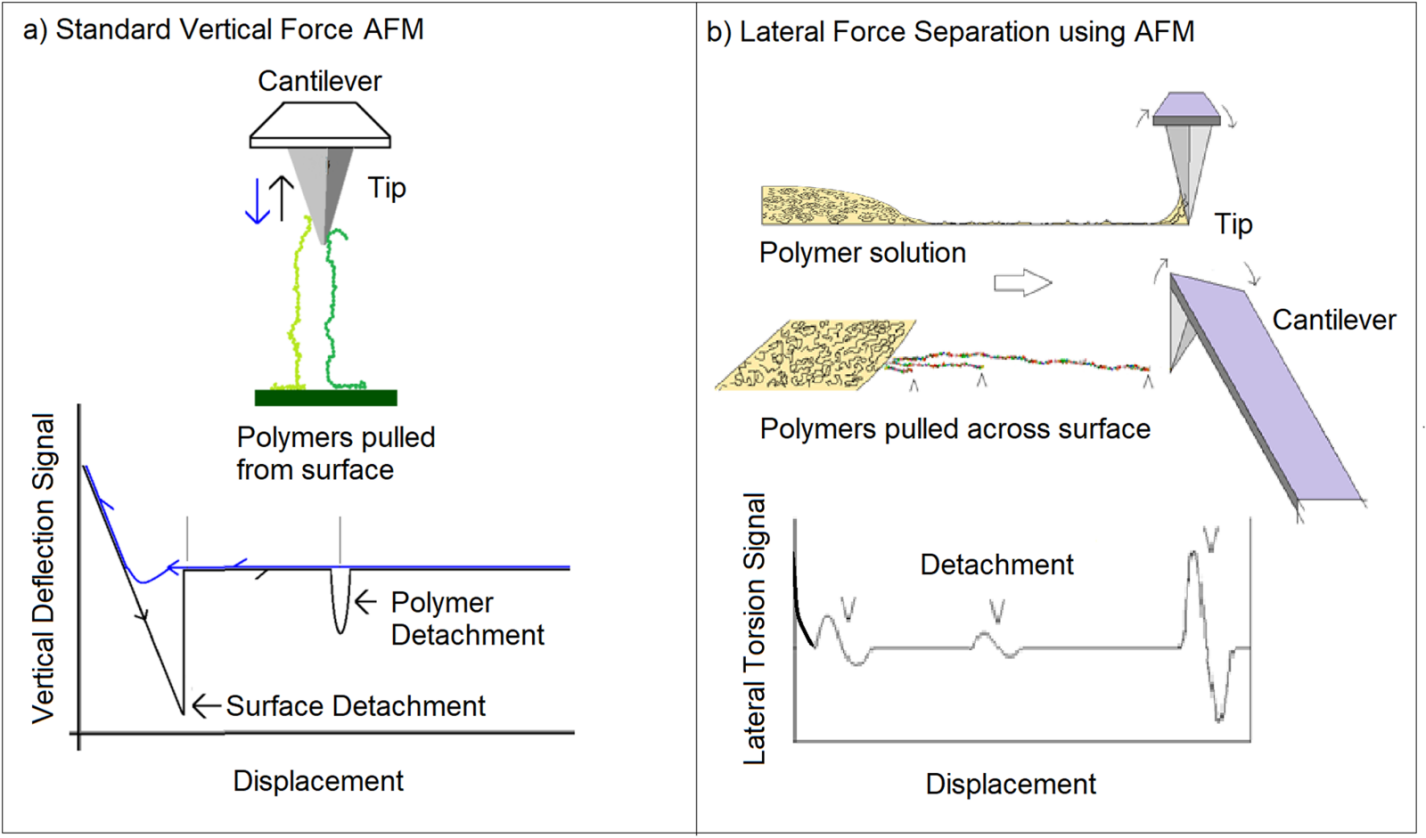
In conventional AFM force spectroscopy, (a) molecular length is measured by pulling a molecule, attached to the surface, off the surface. The new LSF-AFM method (b), a sample solution is deposited on a surface. The edge of the solution is scanned using an AFM in the lateral force detection mode. The lateral (torsional) detachment forces are detected revealing the molecular length.

In this work, a new nanofluidic AFM methodology is presented that is an outgrowth of lateral force AFM.^17^ The new method is termed lateral force separation AFM (LFS-AFM). LSF-AFM mediates molecular separation of molecules onto the AFM tip that is scanned over the edge of a solution deposited on a surface. With LFS-AFM, the tip pulls long chain molecules from the edge of a liquid sample. The lateral force is measured by the torsion on the cantilever detected by the AFM. This produces a characteristic force signal that is indicative of the molecular unwinding and molecular length of the separated molecule upon detachment. Figure 1 illustrates this concept. In contrast to the vertical force spectroscopy, samples remain in a thin film solution and in this sense, it is geometrically similar to AFM mediated, shear driven, liquid chromatography.^18^ The distinct advantage of LFS-AFM is that the surface adhesion stabilizes the detached molecule and serves to tether the molecule so the full length of the molecule can be interrogated—without chemically attaching one end of the molecule to the surface.

The lateral force, shear separation of biopolymers was first tested using a complex, aqueous protein solution. Egg albumen was selected because it was a well characterized complex mixture of proteins. The sample was used, as is, out of the egg shell, undiluted and deposited onto a silicon wafer sample with a glass rod and immediately scanned. The next sample tested was a synthetic DNA using a 20 000 base pair fragment that was analyzed in a buffer solution.

## EXPERIMENTAL

The AFM instrument, a Bruker Icon system (Santa Barbara CA), was operated in a lateral force imaging mode with the scan orientation set to 90°. The deposited sample edge was targeted using the AFM optical microscope as a guide and it was engaged at the sample edge and scanned using 5 × 5 *μ*m image sizes. A molecular detachment signal is obtained using the torsional deflection signal from the AFM cantilever sensor. This system uses a laser focused on the cantilever reflected onto a 4-quadrant photodetector. This photodetector deflection signal is given in mV and recorded in the AFM software as a function of displacement given in nanometers (nm). Note that this signal is used to detect changes in lateral force on the cantilever from molecular detachment as a function of molecular distance. Since this is only used to detect detachment, it is not calibrated for absolute torsion or friction.

‘A silicon AFM tip was used that was obtained from Nanosensors (Neuchatel, Switzerland). This was a PP-LFMR-10, 1–57 kHz with a force constant of approximately 0.2 N/M. The AFM was operated in a contact mode with a set point of 0 V using a 2.6 V off set. This is a standard cantilever off set parameter used in the contact mode for the Bruker software. The scan rate was set to 1 Hz and was not further adjusted. The scan orientation for typical imaging scans is parallel (0°) to the cantilever length. In order to maximize the torsion signal of the cantilever, the scan orientation is set to 90° in the Bruker Icon software. This is perpendicular to the cantilever lengthwise orientation.

The eggs were chicken, Gallus gallus domesticus, obtained from a local market. The egg albumin was used without purification of further analysis. Egg albumin is approximately 8% by weight protein residues.^19^ The DNA fragment was a 20 000 bp provided in a 0.5 *μ*g/*μ*l, in a buffer of 10 mM Tris-HCl buffer, 1 mM EDTA, (pH 7.6), item No. SM1541from NoLimits, Thermo Scientific (Waltham, MA).

## RESULTS

### Egg albumin

**FIG. 2.**
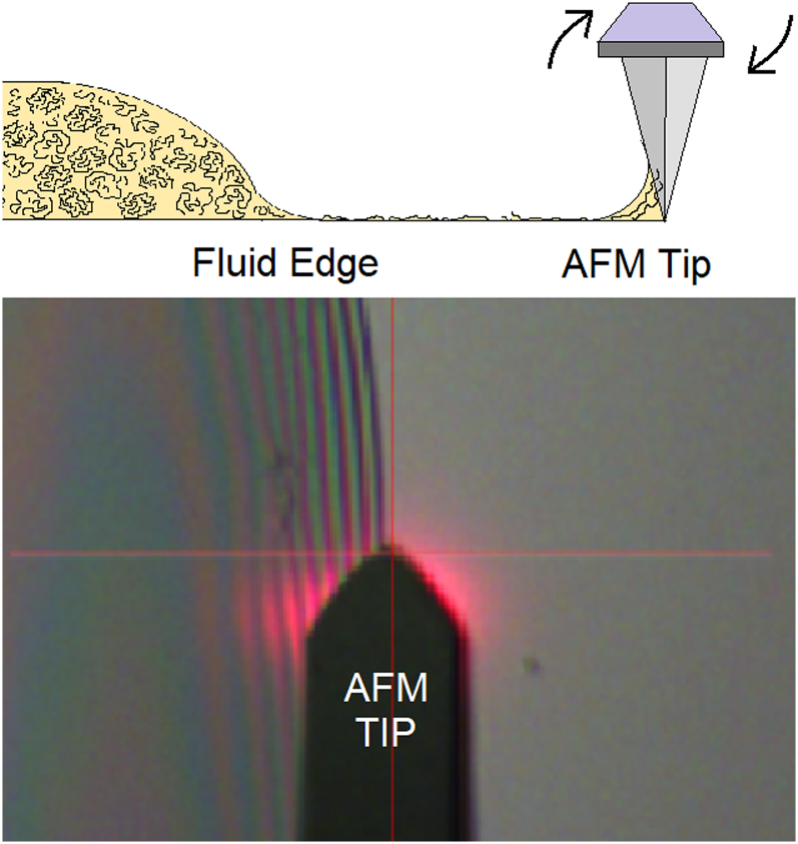
A top view from the AFM's optical viewing microscope photograph. This shows the sample edge and the AFM tip (looking top down). The AFM cantilever is 50 *μ*m wide for scale. As the AFM is scanned over the edge of the sample it pulls polymer strands away from the edge and detects their unfolding and detachment as a torsional, lateral force signal.

**FIG. 3.**
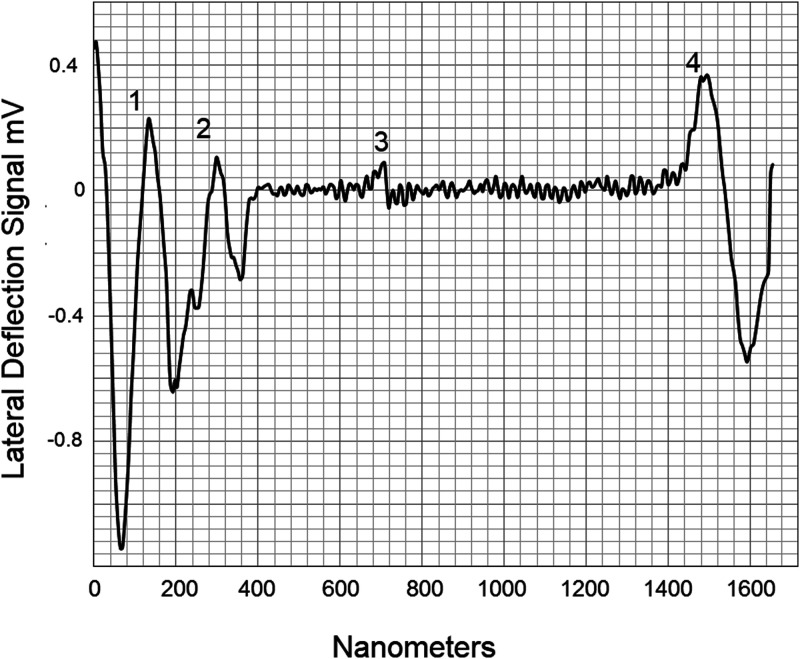
Lateral shear separation of albumen proteins 1. Ovalbumin, 2. Ovotransferrin, 3. Ovomucin, and 4. β-ovomucin. The detachment is apparent in the lateral force signal of the AFM detector. The detachment is detected as a torsional, lateral deflection force signal (mV). The detachment is seen as a torsional force spike followed by a negative peak as the cantilever springs back.

Figure 2 shows the AFM's optical microscope image of egg albumin deposited on a silicon wafer. The AFM was targeted to engage near the solution edge and the scan with a cantilever orientation was set to 90°, in order to maximize the torsional signal. The detachment is detected as a torsional, lateral force signal given in millivolts (mV). Figure 3 shows a single line scan of the torsional force distance plot as the tip is pulled away from the edge of the solution. This reveals a series of long chain strands in the 100–1600 nm length range. The detachment is seen as a torsional force spike followed by a negative peak as the cantilever springs back. The lateral force detachment signal for the separated proteins corresponds to the calculated molecular contour length of the protein where each amino acid monomer contributes 0.38 nm to the length of a polypeptide.^20^ The peaks correspond to the stretched length of the protein as it is pulled from the deposited fluid edge. The approximate protein lengths are shown below:

(1) Ovalbumin 385 amino acids 45 kDa, 54%, calculated length 155 nm.^21^
(2) Ovotransferrin ∼686 amino acids, 76 kDa, 12%, calculated length 266 nm.^22^
(3) α-Ovomucin, ∼2309 amino acids, 254 kDa, 11%, calculated length 877 nm.^23^
(4) β-Ovomucin, 3636–5545 amino acids, 400–610 kDa 11%, calculated length 1381–2107 nm.^23^

### Lateral force separation of DNA

**FIG. 4.**
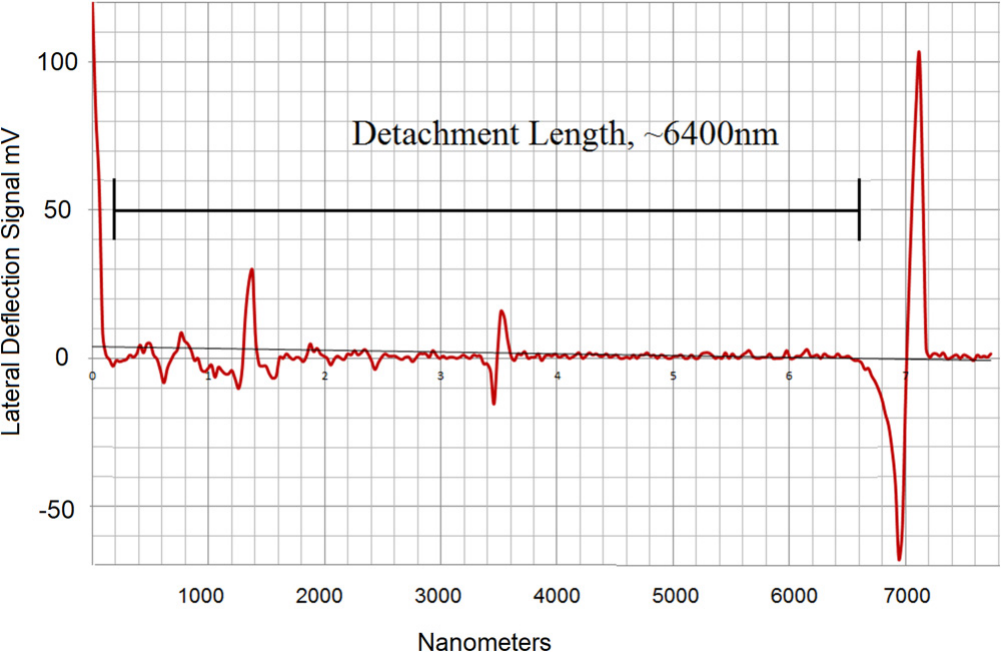
Lateral shear separation of 20 Kbp DNA fragment with a calculated contour length of 6400 nm. The main peak corresponds to the stretched length of the DNA fragment as it is detached from the edge. The detachment is detected in the lateral force signal (mV). The detachment appears as a force spike followed by a negative peak as the cantilever springs back.

A synthetic DNA strand was analyzed in an approach similar to the egg albumin separation (Fig. 4**)**. The sample selected was a 20 000 base pair fragment in a solution of 0.10 mm Tris-HCl buffer and 1 mm EDTA, (pH 7.6). This solution was deposited on a silicon wafer using a micro-syringe and the edge was profiled in the lateral force mode. The DNA fragment has a detachment length that is consistent with the contour length that was calculated using the 0.34 nm per base pair as determined by x-ray diffraction.^24^ The dominant signal corresponds to a molecular length of ∼6400 nm that is measured in the lateral force separation and detachment signal from the edge of the deposited sample. Relatively small peaks are seen at shorter lengths, and these can be due to molecular unwinding, impurities or partial attachment.

## DISCUSSION

The lateral separation of the long chain polymeric strands from the edge of the solution is analogous to the signal observed in other force microscopy measurements, where the AFM tip pulls off molecules vertically from a surface.^9^ In these previous force microscopy experiments, the molecule needs to be chemically bonded with one end tethered to the surface, in order to measure molecular length. An advantage of the LFS-AFM method presented is that the molecule is absorbed on the surface and in the solution to effectively stabilize the molecular strand. This allows the full length of a long chain molecule to be interrogated. The abrupt detachment seen in the force curves is suggestive that the molecular detachment is from the tip. The detachment signal from the fluid edge could be dampened by the length of the polymer. The viscosity of the solutions was not measured directly, these are estimated to be in the range of 0.030–0.018 Pa s for the egg albumin and somewhat lower for the dilute buffered, DNA solution.^25^

The LFS-AFM is not performed as an imaging mode of AFM operation, the AFM is engaged to the surface and operated in a single line scan mode. Consequently, LFS-AFM acquires data relatively fast relative to imaging a surface and is amenable to integration of microfluidic sampling and pre-processing of microscopic samples. It is possible to determine the molecular length by imaging molecules directly using AFM.^26^ However, obtaining molecular resolution AFM images requires a more involved sample preparation and typically purified samples that are deposited on a surface in the proper orientation. The LFS-AFM in contrast, interrogates very complex mixtures without extensive sample preparation. The LFS-AFM method requires very minimal sample preparation—as demonstrated here, fluids with complex mixtures were deposit on a surface “as is” and analyzed immediately after deposition.

## CONCLUSION

The use of LFS-AFM provides the following unique combination of capabilities:

(1) Molecular length without chemical tethering to a surface.
(2) Complex solutions with simple sample preparation can be analysed.
(3) Separation of long chain polymers directly from a solution.
(4) Extremely sensitive detection afforded by the AFM.
(5) Amenable to microfluidic integration.
(6) Small and compact instrumentation.

An AFM is demonstrated here to provide detection of long chain molecules from solutions deposited on surfaces. Long-chain molecules are a general class of biomarkers that has been proposed in life detection.^16,27–29^ This includes proteins, nucleotides, polysaccharides, and extra-cellular bio-films, in general. This work demonstrates a new application of the AFM that is relevant to astrobiology and spacecraft missions.

AFM's have flown on two spacecraft missions, the Phoenix MECA experiment and the Rosetta MIDAS experiment. The implementation of AFM instruments equipped with lateral force spectroscopy on future spacecraft missions may use these existing designs with relatively minor modifications to enable lateral force detection. For implementation, the AFM module would need to be coupled to a microfluidic extraction system similar to what has been proposed for other microfluidic based planetary instruments.^30,31^ Finally, LFS-AFM in a laboratory is planned for the analysis of fossils and meteorites.

## Data Availability

The data that support the findings of this study are available within the article.
